# Enzalutamide Versus Abiraterone plus Prednisolone Before Chemotherapy for Castration-resistant Prostate Cancer: A Multicenter Randomized Controlled Trial

**DOI:** 10.1016/j.euros.2022.04.016

**Published:** 2022-05-19

**Authors:** Kouji Izumi, Takashi Shima, Koji Mita, Yuki Kato, Manabu Kamiyama, Shogo Inoue, Nobumichi Tanaka, Seiji Hoshi, Takehiko Okamura, Yuko Yoshio, Hideki Enokida, Ippei Chikazawa, Noriyasu Kawai, Kohei Hashimoto, Takashi Fukagai, Kazuyoshi Shigehara, Shizuko Takahara, Yoshifumi Kadono, Atsushi Mizokami

**Affiliations:** a Department of Integrative Cancer Therapy and Urology, Kanazawa University Graduate School of Medical Science, Kanazawa, Japan; b Department of Urology, Toyama Prefectural Central Hospital, Toyama, Japan; c Department of Urology, Hiroshima City Asa Citizens Hospital, Horoshima, Japan; d Department of Urology, Fukui-ken Saiseikai Hospital, Fukui, Japan; e Department of Urology, University of Yamanashi, Yamanashi, Japan; f Department of Urology, Institute of Biomedical and Health Sciences, Hiroshima University, Hiroshima, Japan; g Department of Urology, Nara Medical University, Nara, Japan; h Department of Urology, Fukushima Medical University, Fukushima, Japan; i Department of Urology, Anjo Kosei Hospital, Anjo, Japan; j Nephro-Urologic Surgery and Andrology, Mie University Graduate School of Medicine, Tsu, Japan; k Department of Urology, Graduate School of Medical and Dental Sciences, Kagoshima University, Kagoshima, Japan; l Department of Urology, Kanazawa Medical University, Kahoku, Japan; m Department of Nephro-urology, Nagoya City University Graduate School of Medical Sciences, Nagoya, Japan; n Department of Urology, School of Medicine, Sapporo Medical University, Sapporo, Japan; o Department of Urology, Showa University Koto Toyosu Hospital, Tokyo, Japan; p Department of Urology, Ishikawa Prefectural Central Hospital, Kanazawa, Japan; q Innovative Clinical Research Center, Kanazawa University, Kanazawa, Japan

**Keywords:** Androgen deprivation therapy, Androgen receptor signaling–targeted therapy, Endocrine therapy, Castration-resistant prostate cancer, Enzalutamide, Abiraterone, Randomized controlled trial

## Abstract

**Background:**

Enzalutamide (ENZ) and abiraterone plus prednisolone (ABI) improve survival in castration-resistant prostate cancer (CRPC). However, which agent is better for patients with CRPC remains unclear.

**Objective:**

To evaluate whether ENZ or ABI is better as first-line treatment for CRPC.

**Design, setting, and participants:**

An investigator-initiated, multicenter, randomized controlled trial was conducted in Japan. The study enrolled 203 patients with CRPC before chemotherapy between February 20, 2015, and July 31, 2019. Patients were randomly assigned 1:1 to the ENZ or ABI arm.

**Outcome measurements and statistical analysis:**

The primary endpoint was time to prostate-specific antigen (PSA) progression. Secondary endpoints included the PSA response rate (≥50% decline from baseline), overall survival, and safety. A log-rank test was used for comparison of survival analyses between arms.

**Results and limitations:**

After randomization, 92 patients in each arm were treated and analyzed. Time to PSA progression did not significantly differ between the arms (median 21.2 mo for ENZ and 11.9 mo for ABI; hazard ratio [HR] 0.81, 95% confidence interval [CI] 0.51–1.27; *p* = 0.1732). There was a significant difference in the PSA response rate between the arms (72% for ENZ and 57% for ABI; *p* = 0.0425). There was no significant difference in overall survival (median 32.9 mo for ENZA and 35.5 mo for ABI; HR 1.17, 95% CI 0.72–1.88; *p* = 0.5290). Grade ≥3 adverse events were observed in 11% of patients in the ENZA arm and 21% in the ABI arm (*p* = 0.1044).

**Conclusions:**

ENZ did not show any survival benefit in comparison to ABI, but showed a better PSA response rate with a low rate of severe adverse events in CRPC.

**Patient summary:**

Results from our study suggest that use of enzalutamide before abiraterone may have potential clinical benefits for patients with castration-resistant prostate cancer.

## Take Home Message

Enzalutamide (ENZ) and abiraterone plus prednisolone (ABI) improve survival in castration-resistant prostate cancer (CRPC). However, which agent is better for CRPC patients remains unclear. The enzalutamide versus abiraterone plus prednisolone before chemotherapy for castration-resistant prostate cancer study (ENABLE study for PCa) is a head-to-head comparison between ENZ and ABI in this population. ENZ did not show any further survival benefit in comparison to ABI, but showed a better prostate-specific antigen response rate with a low rate of severe adverse events, suggesting that use of ENZ before ABI has potential clinical benefits in CRPC.

## 1 Introduction

Prostate cancer is the most common malignancy and the second leading cause of mortality for males in the USA [1]. The number of PCa patients in Asia, including Japan, has also been increasing [2], [3]. Androgen deprivation therapy (ADT) is the standard treatment for patients with advanced PCa, since its progression is mediated by androgen receptor signaling [4], [5]. However, PCa often progresses to castration-resistant PCa (CRPC), a state characterized by acquired ADT resistance after several years of ADT [6].

Enzalutamide (ENZ) and abiraterone plus prednisolone (ABI), new androgen receptor signaling–targeted agents (ARSTs), improve radiographic progression-free survival (rPFS) and overall survival (OS) in comparison to placebo in metastatic CRPC both before and after docetaxel treatment [7], [8], [9], [10]. ENZ competitively binds to the ligand-binding domain of the androgen receptor and inhibits androgen receptor translocation to the cell nucleus [7]. Abiraterone is a strong inhibitor of CYP17A1, a critical enzyme in androgen synthesis [11]. These oral agents target androgen receptor signaling and are thought to be less toxic than docetaxel. Docetaxel induces more severe neutropenia in the Asian population than in patients from other ethnic backgrounds [12]. Therefore, ENZ and ABI are now widely used as standard first-line therapies for metastatic CRPC in Japan.

ENZ also improves metastasis-free survival and OS in nonmetastatic CRPC, but evidence of a survival benefit with ABI in this setting has not been shown yet [13]. These agents show cross-resistance with each other because of a similar antitumor mechanism [14], [15]. Optimal sequencing of ENZ and ABI was investigated in Canada, with results suggesting that ENZ following ABI was a better sequence for metastatic CRPC. However, no prospective randomized trials have investigated the priority of these agents as single agents rather than for sequential use in metastatic CRPC or in nonmetastatic CRPC. It has been reported that Asians have oncologically different predicted life expectancy in comparison to individuals from other ethnic backgrounds [16]. In addition, CRPC patients are generally older owing to the late onset of PCa and thus have an oncogenic background and may have multiple comorbidities and previous histories. Therefore, determination of which agent is better for initial use in CRPC patients in real-world clinical practice is important. Hence, the ENZ versus ABI before chemotherapy for CRPC study (ENABLE study for PCa) involved a head-to-head comparison between ENZ and ABI as first-line endocrine therapy before chemotherapy for Japanese patients with CRPC, regardless of metastatic status.

## 2 Patients and methods

### 2.1 Study design

The ENABLE study for PCa is an investigator-initiated, phase 3, multicenter, randomized controlled trial in Japan involving a head-to-head comparison of ENZ and ABI for CRPC before chemotherapy. Eligible patients were randomly assigned 1:1 to ENZ 160 mg/d (four 40-mg tablets once a day) or to ABI 1000 mg/d (four 250-mg tablets once a day) and 5 mg of prednisolone twice a day through the data center at the Innovative Clinical Research Center, Kanazawa University, Kanazawa, Japan. Information regarding the patient inclusion and exclusion criteria, random allocation, and data collection methods is provided in the Supplementary material.

This study was conducted in accordance with Ethical Guidelines for Medical and Health Research Involving Human Subjects and the Declaration of Helsinki 1975 (revised in 2013). All treatments and examinations for PCa were undertaken after written informed consent was obtained from each patient before registration. The study received initial approval from the Medical Ethics Committee of Kanazawa University (reference no. 2014-031) and subsequently from the institutional ethics committee of all other participating 15 hospitals (listed in the Supplementary material). The trial was also registered with the University Hospital Medical Information Network (center identifier UMIN000015529) on November 1, 2014.

### 2.2 Definition of endpoints

The primary endpoint was the time to prostate-specific antigen (PSA) progression (TTPP) defined according to the Prostate Cancer Working Group 2 (PCWG2) criteria [6] and outlined below. For patients with a PSA decline at week 13, the PSA progression date was defined as the date on which a ≥25% increase and an absolute increase of ≥2 ng/ml above the nadir were documented. This increase was confirmed by a second consecutive measurement at least 3 wk later. For patients without a PSA decline at week 13, the PSA progression date was defined as the date on which a ≥25% increase and an absolute increase of ≥2 ng/ml above baseline were documented. This was confirmed by a second consecutive measurement at least 3 wk later. The PSA progression date was also defined as the date on which treatment was discontinued if this occurred before week 13 for patients without a PSA decline. For all patients, TTPP was defined as the time from randomization to first confirmed PSA progression. Definitions for the secondary endpoints are included in the Supplementary material.

### 2.3 Statistical analyses

The sample size was calculated on the basis of a study duration of 5 yr and the difference in TTPP between the ENZ and the ABI arms, as previously described [17]. As detailed in the Supplementary material, assuming median TTPP of 11.2 and 7.1 mo in the ENZ and ABI arms, respectively, 91 patients in each arm were required to detect a significant difference between the arms with a two-sided log-rank test at a significance level of 0.05, power of 80%, and hazard ratio (HR) of 0.63 on the basis of previous studies on ENZ and ABI before chemotherapy [7], [8], [18]. The target sample size was set at 100 patients per arm (total of 200 patients) given the assumption that approximately 10% of randomized patients would not be evaluable for various reasons. Intention-to-treat analyses were performed and survival curves were estimated using the Kaplan-Meier method. A log-rank test was used to test for differences in survival between the two arms. HRs were estimated using Cox proportional-hazard models. The PSA response rate was compared between the arms using Fisher’s exact test. All patients were evaluated for toxicity, and the incidence and proportion of grade ≥3 adverse events (AEs) were compared between the arms using Fisher’s exact test. All tests were two-sided, and a *p* value of 0.05 was considered statistically significant.

## 3 Results

**Fig. 1:**
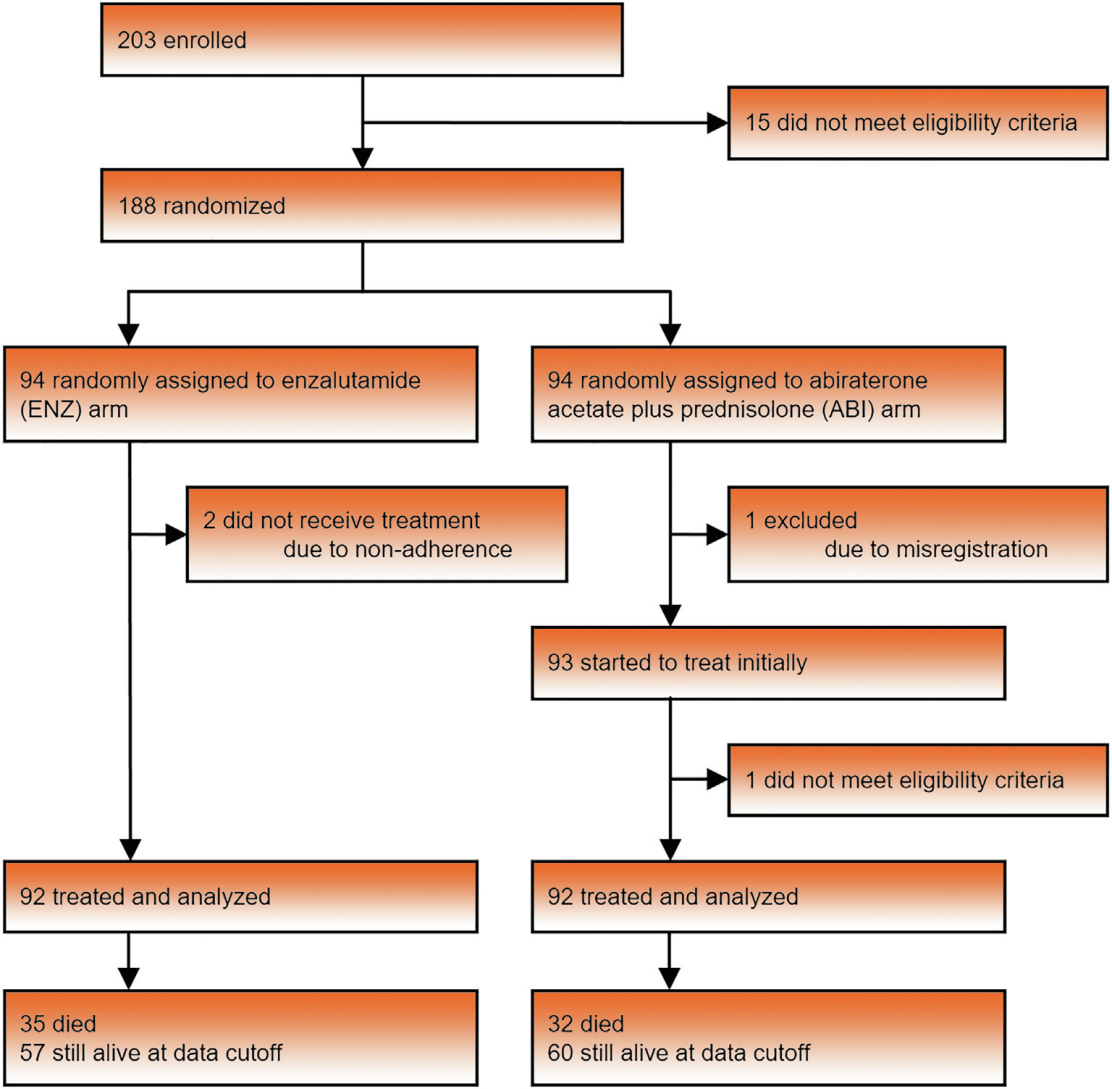
Trial flowchart.

The study enrolled 203 patients from February 20, 2015 to July 31, 2019, with 188 patients randomly assigned to the ENZ or ABI arm (94 patients each) across 16 sites in Japan (Fig. 1). After randomization, 92 patients in the ENZ arm and 92 in the ABI arm were treated and analyzed. The data at the cutoff date of April 22, 2020 were analyzed at median follow-up of 21.5 mo. At the cutoff date, 35 deaths in the ENZ arm and 32 in the ABI arm were confirmed. Table 1 summarizes the baseline characteristics at randomization. Bicalutamide (95% in each arm) and flutamide (59% in the ENZ arm and 55% in the ABI arm; Supplementary Table 1) were used as previous prostate cancer treatments. This investigator-initiated study was conducted within real-world clinical practice and included patients with a wide range of multiple histories and comorbidities (hypertension, diabetes mellitus, and hyperlipidemia were the most frequent) and relatively high age, as expected (Supplementary Tables 2 and 3).

**Table 1:** Baseline characteristics at randomization

Parameter a	ENZ (*n* = 92)	ABI (*n* = 92)	Total (*n* = 184)
Age (yr)	75.7 (70.2–80.4)	77.4 (71.8–81.5)	76.3 (71.0–81.1)
Performance status, *n* (%)			
0	71 (77)	66 (72)	137 (74)
1	19 (21)	23 (25)	42 (23)
Not available	2 (2)	3 (3)	5 (3)
Gleason score, *n* (%)			
5–6	3 (3)	1 (1)	4 (2)
7	9 (10)	15 (16)	24 (13)
8	17 (18)	22 (24)	39 (21)
9	53 (58)	42 (46)	95 (52)
10	9 (10)	8 (9)	17 (9)
Not available	1 (1)	4 (4)	5 (3)
Local treatment, *n* (%)			
Prostatectomy	5 (5)	5 (5)	10 (5)
Irradiation b	22 (24)	18 (20)	40 (22)
None	65 (71)	69 (75)	134 (73)
Regional lymph node metastasis, *n* (%)			
Yes	36 (39)	29 (32)	65 (35)
No	56 (61)	63 (68)	119 (65)
Distant metastasis, *n* (%)			
Bone	62 (67)	63 (68)	125 (68)
Lymph node	23 (25)	13 (14)	36 (20)
Lung	8 (9)	8 (9)	16 (9)
Liver	2 (2)	1 (1)	3 (2)
Other	1 (1)	1 (1)	2 (1)
None	15 (16)	26 (28)	41 (22)
Previous systemic therapies (*n*) c	2.5 (2.0–3.0)	3.0 (2.0–3.0) d	3.0 (2.0–3.0)
Previous bone-modifying agent, *n* (%)			
Yes	9 (10)	9 (10)	18 (10)
No	83 (90)	80 (87)	163 (89)
Not available	0 (0)	3 (3)	3 (2)
Prostate-specific antigen (ng/ml)			
At diagnosis	108.3 (32.4–421.2)	102.4 (19.4–407.4) e	104.5 (30.0–407.8)
At nadir before registration f	0.28 (0.02–1.84)	0.44 (0.04–2.08)	0.33 (0.03–1.93)
At registration	7.5 (3.7–26.16)	11.1 (5.1–21.3)	9.1 (4.2–23.5)
Time from PCDx to randomization (mo) g	30.6 (14.1–63.8)	25.8 (14.9–59.9)	28.5 (14.3–61.3)
Time from CR to randomization (mo) h	1.4 (0.4–5.7)	1.3 (0.4–5.6)	1.4 (0.4–5.6)

ENZ = enzalutamide; ABI = abiraterone plus prednisolone; PCDx = prostate cancer diagnosis; CR = castration resistance.

a Data for continuous variables are presented as median (interquartile range).

b Including high- and low-dose–rate brachytherapy and external beam radiation therapy for the primary site.

c Medical or surgical castration is counted as one therapy.

d Data not available for three patients in the ABI group.

e Data not available for one patient in the ABI group.

f Data not available for two patients in the ENZ group and five in the ABI group.

g Data not available for two patients in the ENZ group and one in the ABI group.

h Data not available for five patients in the ENZ group and eight in the ABI group.

**Fig. 2:**
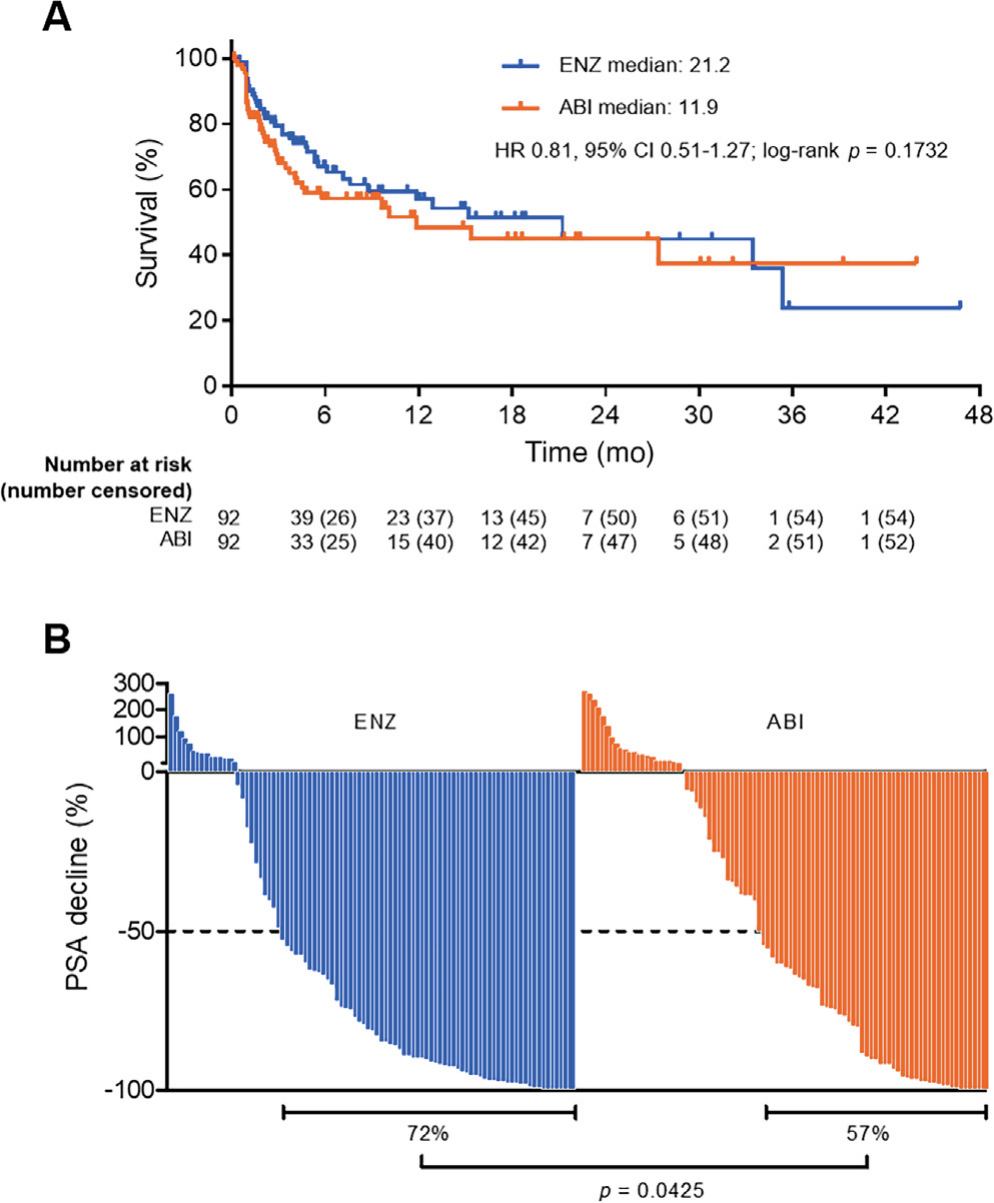
(A) Kaplan-Meier estimate of time to PSA progression and (B) waterfall plot of PSA response. ENZ = enzalutamide; ABI = abiraterone plus prednisolone; PSA = prostate-specific antigen; HR = hazard ratio; CI = confidence interval.

Median TTPP was 21.2 mo in the ENZ arm and 11.9 mo in the ABI arm. The percentage of patients without PSA progression at 6 and 12 mo was 66.9% and 57.0% in the ENZ arm and 57.3% and 48.4% in the ABI arm, respectively. There was no significant difference in TTPP between the arms (HR 0.81, 95% confidence interval [CI] 0.51–1.27; *p* = 0.1732; Fig. 2A). The PSA response rate (≥50% decline in PSA level from baseline) was 72% in the ENZ arm and 57% in the ABI arm (*p* = 0.0425; Fig. 2B).

**Fig. 3:**
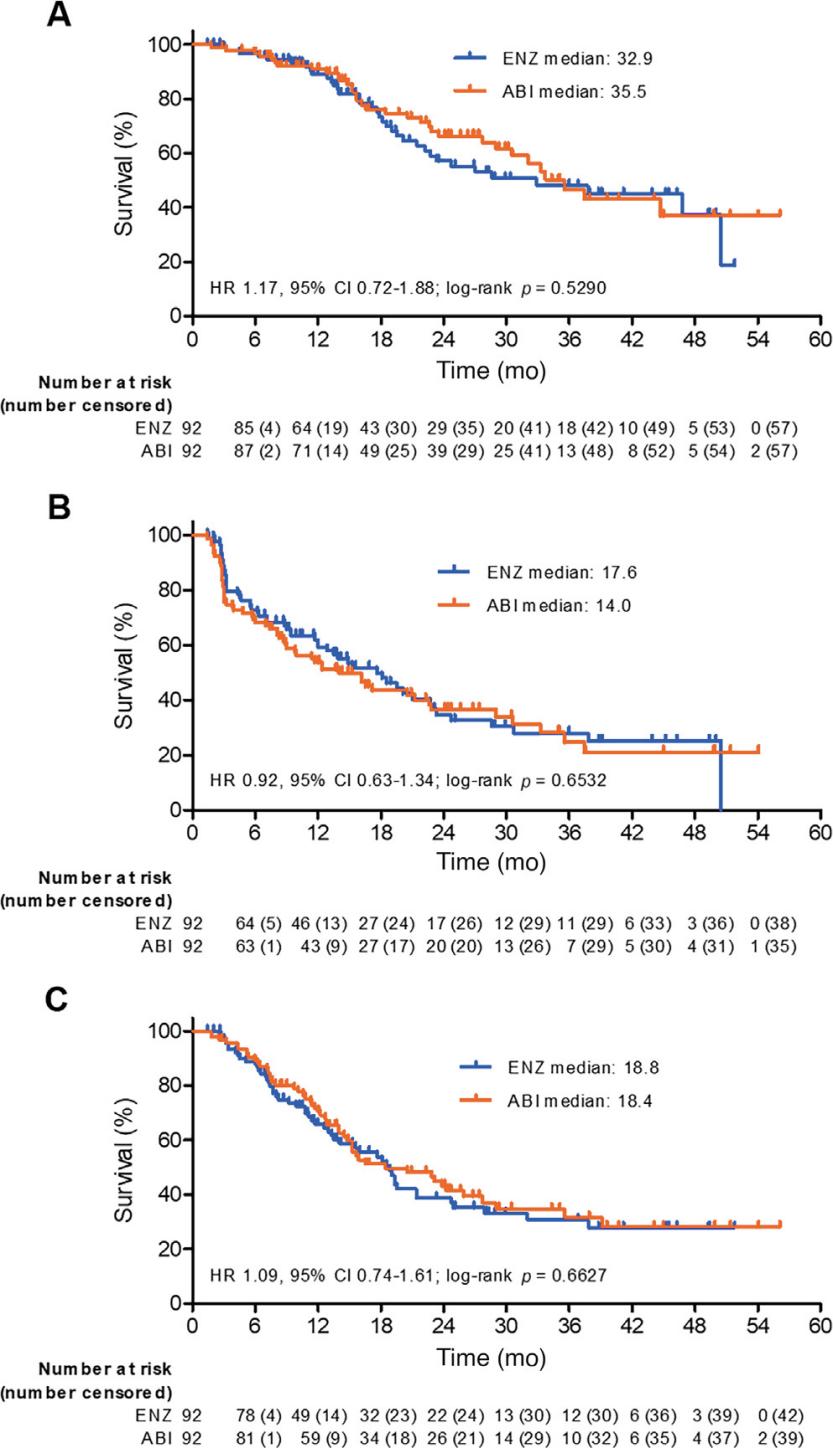
Kaplan-Meier estimates of (A) overall survival, (B) radiographic progression-free survival, and (C) docetaxel-free survival. ENZ = enzalutamide; ABI = abiraterone plus prednisolone; HR = hazard ratio; CI = confidence interval.

Median OS was 32.9 mo in the ENZ arm and 35.5 mo in the ABI arm. The percentage of patients surviving at 6 and 12 mo was 96.6% and 89.0% in the ENZ arm and 96.7% and 90.8% in the ABI arm, respectively. There was no significant difference in OS between the arms (HR 1.17, 95% CI 0.72–1.88; *p* = 0.5290; Fig. 3A). Median rPFS was 17.6 mo in the ENZ arm and 14.0 mo in the ABI arm. The percentage of patients without radiographic progression at 6 and 12 mo was 72.9% and 59.3% in the ENZ arm and 69.4% and 53.8% in the ABI arm, respectively. There was no significant difference in rPFS between the arms (HR 0.92, 95% CI 0.63–1.34; *p* = 0.6532; Fig. 3B).

The best objective response was assessed according to Response Evaluation Criteria in Solid Tumors. The percentage of patients with a complete response, partial response, and stable disease was 2%, 10%, and 52% in the ENZ arm, and 3%, 15%, and 39% in the ABI arm, respectively (Supplementary Table 4).

Median docetaxel-free survival (DFS) was 18.8 mo in the ENZ arm and 18.4 mo in the ABI arm. The percentage of patients without docetaxel treatment at 6 and 12 mo was 87.5% and 65.7% in the ENZ arm and 89.0% and 70.3% in the ABI arm, respectively. There was no significant difference in DFS between the arms (HR 1.09, 95% CI, 0.74–1.61; *p* = 0.6627; Fig. 3C).

Median PCa-specific survival was 46.8 mo in the ENZ arm and 44.7 mo in the ABI arm. The percentage of patients surviving at 6 and 12 mo was 97.7% and 90.0% in the ENZ arm and 96.7% and 90.8% in the ABI arm, respectively. There was no significant difference in PCa-specific survival between the arms (HR 1.24, 95% CI 0.78–2.08; *p* = 0.4227; Supplementary Fig. 1A).

Median performance status progression (PSP)-free survival was 32.9 mo in the ENZ arm and 33.7 mo in the ABI arm. The percentage of patients without PSP at 6 and 12 mo was 88.8% and 85.0% in the ENZ arm and 96.7% and 86.0% in the ABI arm, respectively. There was no significant difference in PSP-free survival between the arms (HR 1.04, 95% CI 0.35–1.60; *p* = 0.8759; Supplementary Fig. 1B).

We also analyzed TTPP in metastatic and nonmetastatic groups separately. The median TTPP for patients with metastatic disease was 15.2 mo in the ENZ arm and 11.9 mo in the ABI arm (HR 0.81, 95% CI 0.49–1.35; *p* = 0.4080; Supplementary Fig. 2A). The median TTPP for patients with nonmetastatic disease was 33.5 mo in the ENZ arm and 27.4 mo in the ABI arm (HR 0.56, 95% CI 0.21–1.50; *p* = 0.2169; Supplementary Fig. 2B).

After the study treatment, 55% of patients in the ENZ arm and 63% in the ABI arm received a subsequent systemic treatment for PCa. For second-line treatment, docetaxel was the most frequent agent (23%) followed by ABI (20%) in the ENZ arm., while ENZ was the most frequent (30%) followed by docetaxel (23%) in the ABI arm. Subsequent treatments for PCa (including rechallenge with the study treatments) were reported up to the seventh line (Supplementary Table 5).

AEs of any cause were observed in 65% of patients in each arm. A grade ≥3 AE was observed for 11% of patients in the ENZ arm and 21% in the ABI arm. Although malaise and digestive symptoms were frequent AEs in the ENZ arm, they were rarely severe. Elevation of liver enzymes was a frequent AE and often became serious in the ABI arm. However, there was no significant difference in the frequency of grade ≥3 AEs between the arms (*p* = 0.1044; Table 2; full information is listed in Supplementary Table 6).

**Table 2:** Adverse events a

	Patients, *n* (%)			
	ENZ (*n* = 92)		ABI (*n* = 92)	
	Any grade	Grade ≥3	Any grade	Grade ≥3
Adverse events of any cause	60 (65)	10 (11)	60 (65)	19 (21)
Anemia	17 (18)	3 (3)	19 (21)	3 (3)
Thrombocytopenia	5 (5)	0	4 (4)	1 (1)
Malaise	22 (24)	0	7 (8)	1 (1)
Fatigue	7 (8)	0	4 (4)	1 (1)
Decreased appetite	16 (17)	1 (1)	9 (10)	1 (1)
Nausea	9 (10)	1 (1)	3 (3)	1 (1)
Body weight loss	7 (8)	0	8 (9)	1 (1)
Elevated aspartate aminotransferase	8 (9)	1 (1)	16 (17)	4 (4)
Elevated alanine aminotransferase	6 (7)	2 (2)	15 (16)	7 (8)
Fracture	2 (2)	2 (2)	4 (4)	2 (2)
Bone pain	3 (3)	2 (2)	6 (7)	3 (3)
Hypertension	3 (3)	0	7 (8)	3 (3)
Edema	1 (1)	0	5 (5)	1 (1)
Rash	2 (2)	0	2 (2)	2 (2)

ENZ = enzalutamide; ABI = abiraterone plus prednisolone.

a Adverse events with a frequency ≥5% for any grade or ≥2% for or grades 3–5 are shown.

## 4 Discussion

This investigator-initiated, multicenter, randomized controlled trial demonstrated a lack of significant differences in TTPP, OS, rPFS, and DFS between ENZ and ABI. However, patients in the ENZ arm experienced a significantly better PSA response rate than those in the ABI arm, in addition to relatively low incidence of severe AEs.

A Canadian group reported that ENZ following ABI is a better treatment sequence for metastatic CRPC from an analysis of a phase 2 crossover trial that included 202 patients [11]. Time to second PSA progression was longer in the ENZ following ABI arm than in the reverse-order arm (median 19.3 vs 15.2 mo; HR 0.66, 95% CI 0.45–0.97; *p* = 0.036) [11]. Moreover, a systematic review revealed that ENZ after ABI led to significantly longer PSA progression–free survival than for ABI after ENZ [19]. Similar results were retrospectively reported for 255 CRPC patients in Japan without chemotherapy. Longer TTPP was observed after second-line ENZ treatment following ABI than after the reverse-order sequence (median 3.67 vs 2.07 mo; HR 0.67, 95% CI 0.51–0.87; *p* = 0.021) [20]. However, no prospective randomized trials have investigated the priority for these agents as single agents rather than for sequential use. It has been reported that Asian patients have oncologically different predicted life expectancy in comparison to individuals from other ethnic backgrounds [16]. Different ethnic backgrounds are expected to show different treatment outcomes, such as the favorable survival reported for Asian patients with PCa treated with ADT in comparison to their Caucasian counterparts [21]. Treatment-related AEs and the optimal dose of such agents also differ by ethnicity because of differences in physical constitution [12], [22], [23].

Sequential treatment with ENZ and ABI is not mandatory because several promising agents with different anticancer mechanisms are currently available (eg, docetaxel, radium-223, and olaparib). Sequential use of these oral ARSTs should rather be avoided for cases for which higher biological effectiveness is required [24]. A prospective head-to-head study reflecting real-world clinical practice was greatly needed to guide ARST treatment decisions because of the paucity of clinical efficacy and safety data for ENZ and ABI for CRPC in the Asian population. The ENABLE study for PCa is the first study of its kind. The aim was to clarify which agent should be prioritized for patients with CRPC to enable clinicians to decide on the most appropriate treatment before chemotherapy.

The PSA response rate was higher in the ENZ arm, although there were no significant differences in any survival outcomes between the arms. These results are similar to findings from previous studies [11], [20]. Interestingly, a recent systematic review and meta-analysis revealed no significant difference in OS improvement between ENZ and ABI, but ENZ was superior to ABI in improving rPFS and TTPP for patients with metastatic CRPC [25]. In addition, a large retrospective analysis of the Veterans Health Administration database revealed that patients treated with ENZ had a 16% lower risk of death in comparison to those treated with ABI (HR 0.84, 95% CI 0.76–0.94; *p* = 0.0012) [26]. These data indicate a stronger ability of ENZ to inhibit androgen receptor signaling as a single agent. The traditional sequence for these ARSTs persisted because of limited treatment choices several years ago, and the time to second PSA progression or second PSA response rate might be valuable in making judgments on more effective use of ENZ and ABI in terms of better quality of life and cost-effectiveness. In fact, only 46 of the 184 patients in our real-world cohort received sequential treatment with these ARSTs. In addition, a wide variety of treatments for PCa, including vintage hormonal therapies (eg, flutamide, ethynilestradiol, and estramustin phosphate), were used after ENZ and ABI, suggesting that many drugs may contribute to patient survival after ENZ or ABI. Sequential treatment with ENZ and ABI may no longer be regarded as a major treatment strategy in real-world clinical practice.

Although there were no significant differences in AEs between the ENZ and ABI arms, high incidence of elevated liver enzymes (aspartate aminotransferase and alanine aminotransferase) in the ABI arm was notable. It has been reported that elevation of liver enzymes typically occurs within the first 2 mo on ABI. Most patients experience normalization, either spontaneously or after dose reduction/discontinuation [27]. However, the high incidence of grade ≥3 elevation of liver enzymes should not be overlooked, as mortality due to fulminant hepatitis after ABI receipt has been reported in Japan [28]. Moreover, a higher incidence of severe hypertension was observed in the ABI arm. The proportions of patients affected are similar to those observed in the COU-AA-302 trial, which was the first to show that ABI improves OS in CRPC [18]. High incidence of malaise, fatigue, and digestive organ toxicities was observed in the ENZ arm; however, most of these cases were not severe. Seizure, which has been reported at a higher rate during ENZ treatment, was observed in only one patient in each arm and was of low grade. These AE profiles for the two arms suggest that more careful attention should be focused on patients treated with ABI than those treated with ENZ in the Asian population.

The current study has several limitations despite a number of strengths. All of the patients were Japanese and patients from other countries were not included. An open-label study has potential biased risks for dose reduction/discontinuation or interpretation of data acquired during the treatment courses. Currently, new ARSTs and docetaxel are available for metastatic castration-sensitive PCa and the number of potential candidates for studies in CRPC may be decreasing in real-world clinical practice. Patients included in the current study were oncologically heterogeneous and the cohort had more than ten types of malignancy in their medical histories. In addition, all comorbidities were allowed except for some predefined states. These factors may decrease the evidential power regarding survival and safety for patients treated with ENZ and ABI for CRPC because wide differences were noted between OS and PCa-specific survival in current study.

## 5 Conclusions

The current study revealed that ENZ did not show any survival benefit in comparison to ABI but showed a better PSA response rate and a low rate of severe AEs in patients with CRPC for the first time. The data suggest that use of ENZ before ABI has potential clinical benefits for patients with CRPC.

  ***Author contributions***: Kouji Izumi had full access to all the data in the study and takes responsibility for the integrity of the data and the accuracy of the data analysis.

*Study concept and design:* Izumi.

*Acquisition of data:* Izumi, Shima, Mita, Kato, Kamiyama, Inoue, Tanaka, Hoshi, Okamura, Yoshio, Enokida, Chikazawa, Kawai, Hashimoto, Fukagai, Shigehara, Takahara.

*Analysis and interpretation of data:* Izumi, Takahara, Mizokami.

*Drafting of the manuscript:* Izumi.

*Critical revision of the manuscript for important intellectual content:* Izumi, Shima, Mita, Kato, Kamiyama, Inoue, Tanaka, Hoshi, Okamura, Yoshio, Enokida, Chikazawa, Kawai, Hashimoto, Fukagai, Shigehara.

*Statistical analysis:* Izumi, Takahara.

*Obtaining funding:* Izumi.

*Administrative, technical, or material support:* Kato, Takahara, Kadono, Mizokami.

*Supervision:* Mizokami.

*Other:* None.

  ***Financial disclosures:*** Kouji Izumi certifies that all conflicts of interest, including specific financial interests and relationships and affiliations relevant to the subject matter or materials discussed in the manuscript (eg, employment/affiliation, grants or funding, consultancies, honoraria, stock ownership or options, expert testimony, royalties, or patents filed, received, or pending), are the following: None.

  ***Funding/Support and role of the sponsor*:** The ENABLE study for PCa received external funding from the Japanese Foundation for Multidisciplinary Treatment of Cancer. This funding was mainly used for software for patient randomization.
