# Supplementary material for: Enzalutamide Versus Abiraterone plus Prednisolone Before Chemotherapy for Castration-resistant Prostate Cancer: A Multicenter Randomized Controlled Trial
Source: Eur Urol Open Sci. 2022 May 19;41:16–23. doi: 10.1016/j.euros.2022.04.016 (PMC9257638; doi:10.1016/j.euros.2022.04.016)

**Supplementary material**

**Patient inclusion and exclusion criteria**

Patients included if (1) have pathologically or cytologically confirmed CRPC, defined as total testosterone levels <50 ng/dL and two consecutive prostate-specific antigens (PSA) elevations with 1 week interval, where the PSA used for judgment is at least 2 ng/mL higher than nadir; (2) have had no previous cytotoxic intravenous systemic chemotherapy; (3) are ≥20 years when providing written informed consent; (4) have a performance status (PS) of 0–2 according to the Eastern Cooperative Oncology Group; (5) have appropriate hepatic and renal functionality as demonstrated in laboratory tests within 4 weeks before registration (total bilirubin level ≤1.5 × upper limit of normal (ULN), aspartate transaminase ≤2.5 × ULN (≤5.0 × ULN in patients with liver metastasis), alanine transaminase ≤2.5 × ULN (≤5.0 × ULN in patients with liver metastasis), and serum creatinine ≤2.0 × ULN and neither ascites nor hepatic encephalopathy are present); and (6) have a life expectancy >3 months. Patients are ineligible if they (1) have an allergy to enzalutamide (ENZ), or abiraterone plus prednisolone (ABI) tereatment; (2) desire to have children; and (3) are considered by a principal or clinical investigator to be inappropriate for participation in the present study for any other reasons. A history of any other treatments for which efficacy had not been shown in a randomized controlled trial at the time of starting the current study was permitted except for cytotoxic intravenous chemotherapies.

The ENZ or ABI administration was terminated when: (1) PSA progression was confirmed, (2) the patient expired, or (3) severe adverse events (AEs) occurred. Luteinizing hormone-releasing hormone agonist (or antagonist) was continued throughout the study. Zoledronic acid and denosumab were permitted for patients with bone metastasis. Any sequential treatments were permitted after the confirmation of PSA progression in both arms. Dose reduction was permitted if a principal or clinical investigator considered the basic doses inappropriate for any reason.

**Random allocation and data collection**

Randomization is centrally performed by an automatic randomization system, Waritsukekun (Mebix, Inc. Tokyo, Japan), using a minimization method to obtain adequate between-arm balance for age category (<70 or ≥70), the Eastern Cooperative Oncology Group PS (0–1/2), the status of metastasis (none, bone alone, or other than bone),and participating institution. Investigators and patients were not masked treatment assignments.All patients providing written informed consent to participate in the study were asked to complete their medical history. Obtained clinical data in the ENABLE study for PCa include PS; physical examination findings; hematological, blood biochemical, and urine test results; chest X-ray imaging; lung-to-pelvic computed tomography (CT) or magnetic resonance imaging (MRI); bone scintigraphy with or without a bone scan index; and treatment information. Chest X-ray and head and neck CT or MRI were performed during study registration. Other examinations were performed every month from the date of commencement to month 6, and every 3 months after month 6 until the study was completed. However, these examinations could be performed at any time if a principal or clinical investigator considered it necessary.

**Institutional Review Board details**

Medical Ethics Committee of Kanazawa University^1^

Research Ethics Committee of Toyama Prefectural Central Hospital^2^

Ethics Committee of Hiroshima City Asa Citizens Hospital^3^

Fukui-Ken Saiseikai Hospital Institutional Review Board^4^

The Research Ethics Committee of Faculty of Medicine, University of Yamanashi^5^

Ethical Committee for Clinical Research of Hiroshima University^6^

Medical Ethics Committee of Nara Medical University^7^

Ethics review committee of Fukushima Medical University^8^

Ethics Committee of Anjo Kosei Hospital^9^

Institutional Review Board, Mie University Hospital^10^

Ethics Committee of Kagoshima University Medical and Dental Hospital^11^

Ethics Committee of Kanazawa Medical University^12^

The Nagoya City University Graduate School of Medical Sciences and Nagoya City University Hospital Institutional Review Board^13^

Institutional Review Board of Sapporo Medical University Hospital^14^

Institutional Review Board, Showa University of Koto Toyosu Hospital^15^

The Ethics Committee of Ishikawa Prefectural Central Hospital^16^

^1^Department of Integrative Cancer Therapy and Urology, Kanazawa University Graduate School of Medical Science, 13-1 Takaramachi, Kanazawa, Ishikawa 920-8641, Japan.

^2^Department of Urology, Toyama Prefectural Central Hospital, 2-2-78 Nishinagae, Toyama 930-8550, Japan.

^3^Department of Urology, Hiroshima City Asa Citizens Hospital, 2-1-1 Kabeminami, Asakita-ku, Horoshima 731-0293, Japan.

^4^Department of Urology, Fukui-ken Saiseikai Hospital, 7-1 Wadanakacho-Funabashi, Fukui, Fukui 918-8503, Japan.

^5^Department of Urology, University of Yamanashi, 1110 Shimokato, Chuo, Yamanashi 409-3898, Japan.

^6^Department of Urology, Institute of Biomedical and Health Science, Hiroshima University, 1-2-3 Kasumi, Minami-ku, Hiroshima, Hiroshima 734-8551, Japan.

^7^Department of Urology, Nara Medical University, 840 Shijocho, Kashihara, Nara 634-8521, Japan.

^8^Department of Urology, Fukushima Medical University, 1 Hikarigaoka, Fukushima, Fukushima 960-1295, Japan.

^9^Department of Urology, Anjo Kosei Hospital, 28 Anjocho-Higashihirokute, Anjo, Aichi 446-8602, Japan.

^10^Nephro-Urologic Surgery and Andrology, Division of Reparative and Regenerative Medicine, Institute of Medical Life Science, Mie University Graduate School of Medicine, 2-174 Edobashi, Tsu, Mie 514-8507, Japan.

^11^Department of Urology, Graduate School of Medical and Dental Sciences, Kagoshima University, 8-35-1 Sakuragaoka, Kagoshima, Kagoshima 890-8520, Japan.

^12^Department of Urology, Kanazawa Medical University, 1-1 Uchinadamachi-Daigaku, Kahoku, Ishikawa 920-0293, Japan.

^13^Department of Nephro-urology, Nagoya City University Graduate School of Medical Sciences, 1 Mizuhocho-Aza-Kawasumi, Mizuho-ku, Nagoya, Aichi 467-8602, Japan.

^14^Department of Urology, School of Medicine, Sapporo Medical University, 16-291 Minami-1-Jo-Nishi, Sapporo, Hokkaido 060-8543, Japan.

^15^Department of Urology, Showa University Koto Toyosu Hospital, 5-1-38 Toyosu, Koto-ku, Tokyo 135-8577, Japan.

^16^Department of Urology, Ishikawa Prefectural Central Hospital, 2-1 Kuratsukihigashi, Kanazawa, Ishikawa 920-8530, Japan.

^17^Innovative Clinical Research Center, Kanazawa University, 13-1 Takaramachi, Kanazawa, Ishikawa 920-8641, Japan.

**Definitions of secondary endpoints**

Secondary endpoints include (1) PSA response rate (≥50% decline in PSA level from baseline); (2) OS defined as the time from randomization to mortality from any cause; (3) rPFS based on the Response Evaluation Criteria in Solid Tumors (RECIST), version 1.1, criteria for soft-tissue lesions examined with computed tomography or magnetic resonance imaging and PCWG2 criteria for bone metastasis examined with bone scintigraphy; (4) time to the commencement of docetaxel; (5) safety according to the frequency and grade using Common Terminology Criteria for Adverse Events (CTCAE), version 4.0 (http://evs.nci.nih.gov/ftp1/CTCAE/About.html); (6) prostate cancer-specific survival defined as the time from randomization to mortality from prostate cancer; and (7) PS progression-free survival. Some prespecified endpoints were not analyzed in this study because full discretion was given to each investigator and heterogeneous second and later line therapies and the lack of data made analyses with statistical reliability difficult.

**Assumption of a median TTPP in the ENZ and ABI arm**

We assumed two patterns of median TTPP of ABI based on results of previous phase 3 studies, PREVAIL for ENZ and COU-AA-302 for ABI (as shown in the figure below).^7, 8^ Pattern 1: If TTPP of control arms of PREVAIL and COU-AA-302 were same and a median TTPP of prednisolone (PSL) arm was 2.8 months, and a median TTPP of ABI was 5.6 months (11.1 × 2.8 / 5.6). Pattern 2: PSL has been reported to have a moderate anti-tumor effect in prostate cancer patients and to extend 2 months in TTPP. As PSL was administered to all patients in the ABI study, TTPP of the ABI treatment group may be reduced to 3.6 months. A median TTPP of ABI was 8.6 months (11.1 × 2.8 / 3.6). We used 7.1 months, the average of 5.6 and 8.6 months, as a median TTPP of ABI.^17^


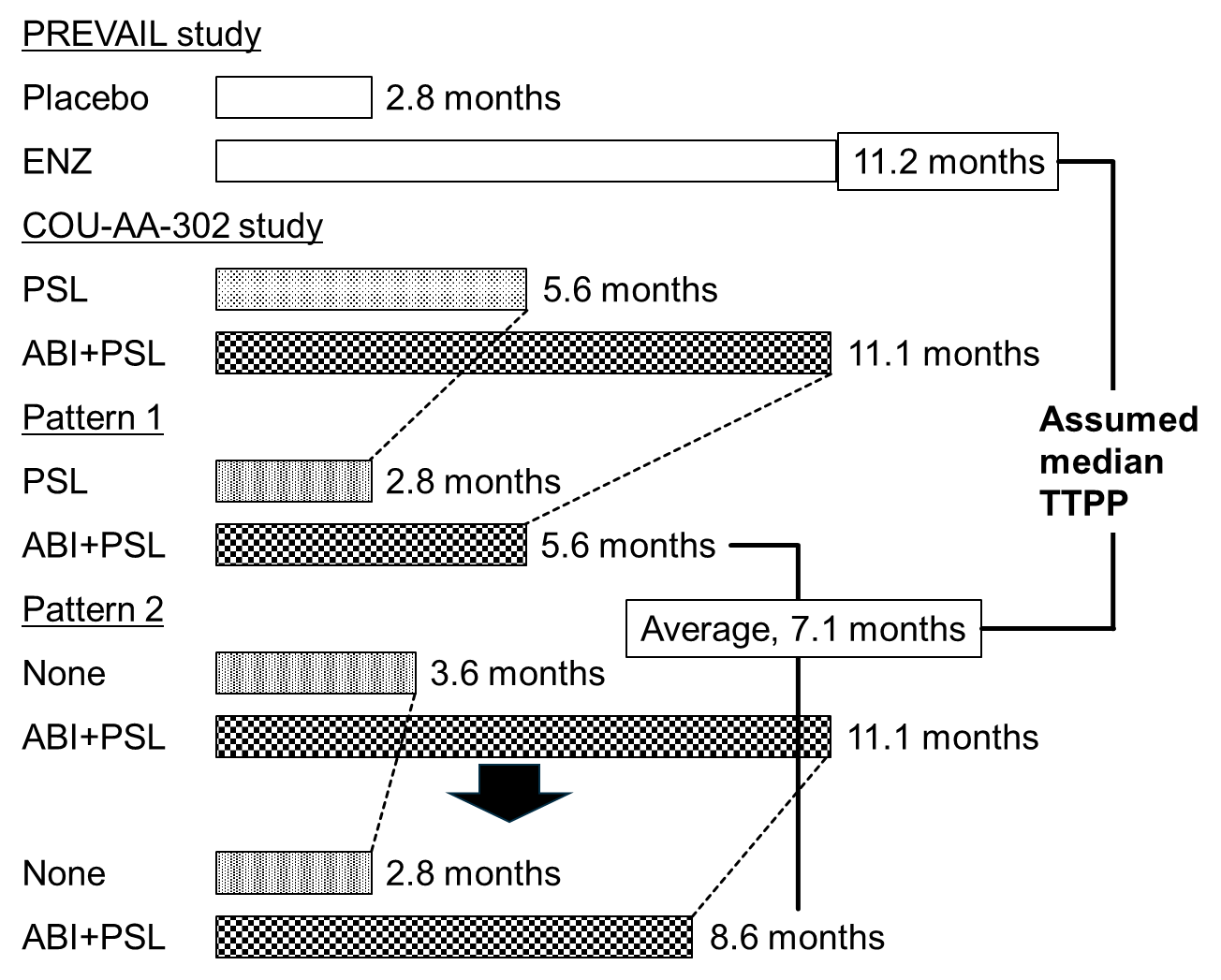

Supplement: Supplementary data 1 [file mmc1.docx]
