# Supplementary material for: Enzalutamide Versus Abiraterone plus Prednisolone Before Chemotherapy for Castration-resistant Prostate Cancer: A Multicenter Randomized Controlled Trial
Source: Eur Urol Open Sci. 2022 May 19;41:16–23. doi: 10.1016/j.euros.2022.04.016 (PMC9257638; doi:10.1016/j.euros.2022.04.016)
Supplement: Supplementary data 2 [file mmc2.pptx]

## Slide 1
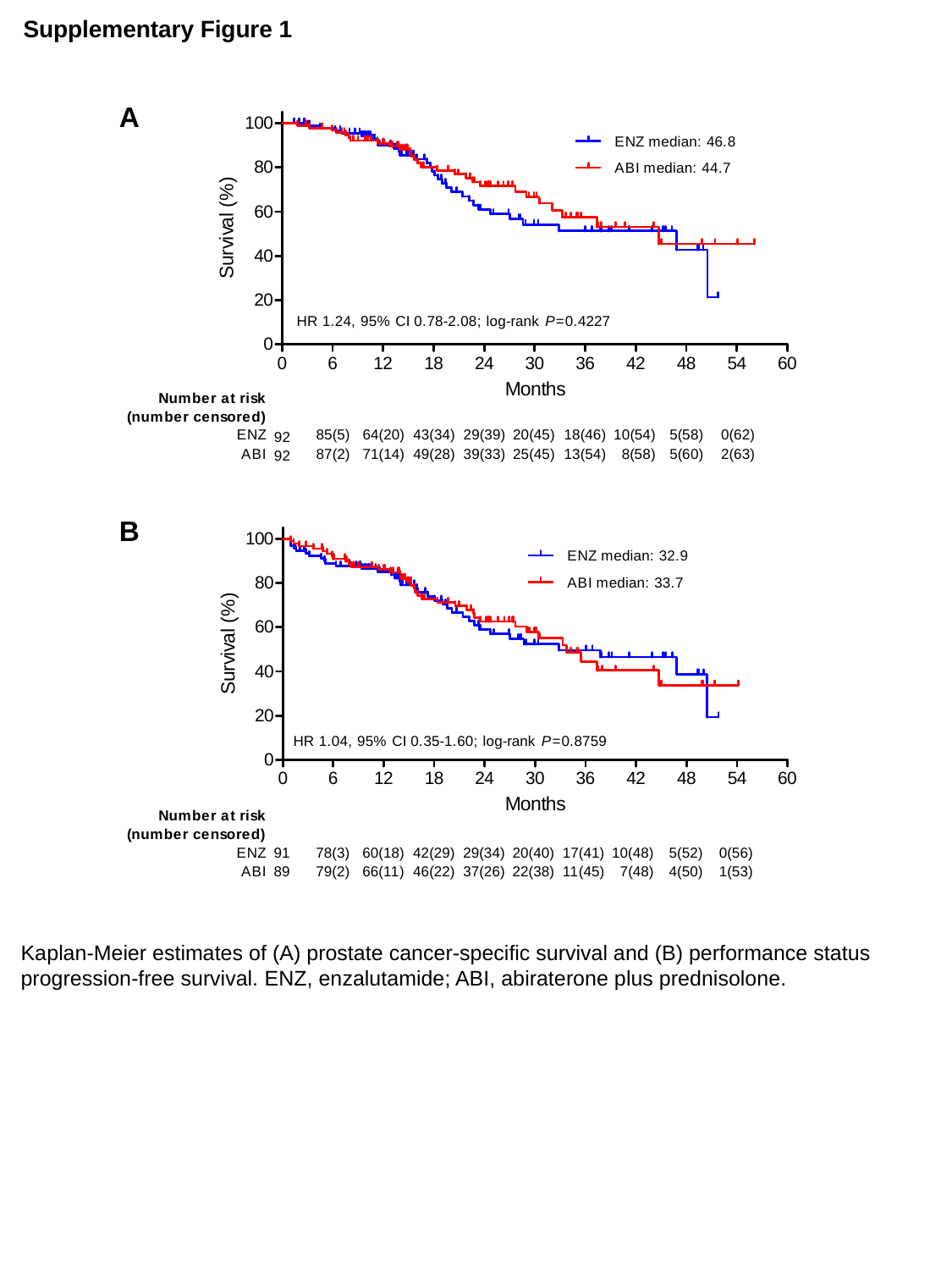

Supplementary Figure 1
A
B
Kaplan-Meier estimates of (A) prostate cancer-specific survival and (B) performance status progression-free survival. ENZ, enzalutamide; ABI, abiraterone plus prednisolone.

## Slide 2
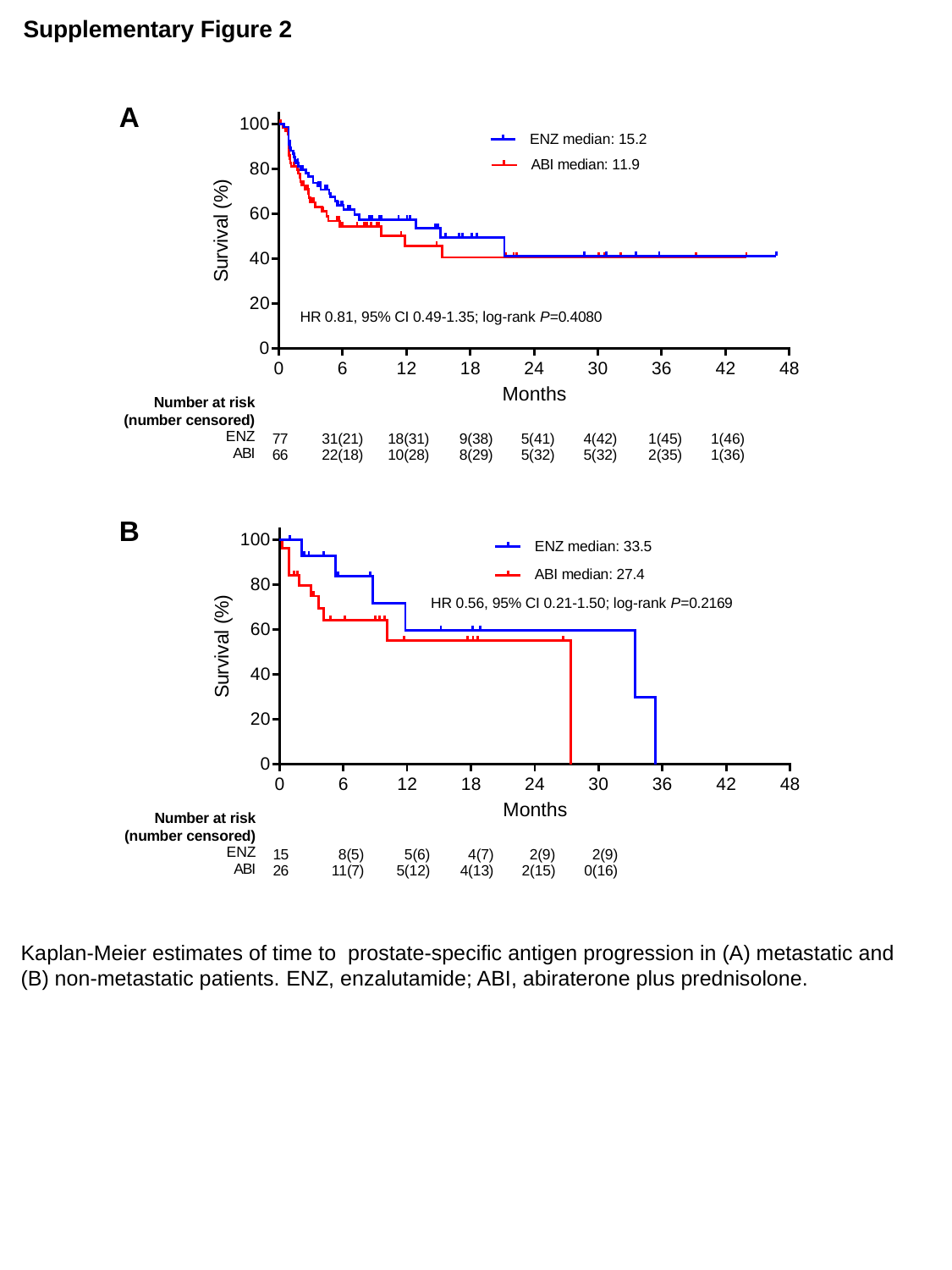

Supplementary Figure 2
A
B
Kaplan-Meier estimates of time to prostate-specific antigen progression in (A) metastatic and (B) non-metastatic patients. ENZ, enzalutamide; ABI, abiraterone plus prednisolone.
